# Strengthening Behavioral Health Services Through Partnerships and Data Integration

**DOI:** 10.7759/cureus.24929

**Published:** 2022-05-11

**Authors:** Shama Faheem, Andrea Smith, Eric Doeh, Robert Dunne, Damon Gorelick

**Affiliations:** 1 Behavioral Health, Detroit Wayne Integrated Health Network, Detroit, USA; 2 Emergency Medicine, Wayne State University, Detroit, USA; 3 Emergency, Detroit East Medical Control Authority, Detroit, USA

**Keywords:** behavioral health, crisis, emergency, ems, diversion

## Abstract

Background

There has been an increase in emergency medical service (EMS) use for behavioral health reasons. Detroit Wayne Integrated Health Network (DWIHN) and Detroit East Medical Control Authority (DEMCA) collaborated to study the rising number of behavioral health (mental disorders and substance use disorders) calls to EMS.

Methodology

To examine the trend, DWIHN and DEMCA partnered on a data-sharing project and identified that a high volume of EMS runs (responses by EMS as a result of an emergency call) involved individuals served by DWIHN.

Results

Over a period of 2.5 years, an average of one-third (33.73%) of EMS runs involved individuals who receive behavioral health services through DWIHN.

Conclusions

DWIHN used the data to create interventions and internal process improvements that can help coordinate medical and behavioral healthcare for individuals who have been using EMS increasingly. The findings were also used to develop prevention efforts to decrease the occurrence of such crises and to avoid unwarranted member involvement with the justice system. We suggest that other comparable organizations consider similar partnerships, especially given the increasingly high EMS and Emergency Department use for behavioral health reasons.

## Introduction

Approximately one in eight visits to emergency departments (EDs) in the United States involves mental and substance use disorders [1]. Between 2007 and 2011, the rate of ED visits related to behavioral health increased by over 15% [2]. Not surprisingly, shifts in emergency room (ER) visits have occurred during the coronavirus disease 2019 pandemic, with an even higher percentage of behavioral health visits when compared to the previous pre-pandemic year [3]. The high number of emergency medical service (EMS) calls coupled with subsequent high ED utilization to address patients’ behavioral health concerns have been well documented [4-7]. Regarding pediatric EMS use, chronic somatic conditions and behavioral health problems appear to contribute to a large proportion of the repeat pediatric EMS use [8].

EMS personnel are often the first contact for individuals in behavioral health crises in the community; almost 14% (almost 7.5 million) of emergency visits for behavioral health conditions from 1992 to 2001 involved EMS transports [9]. According to a study on behavioral health transports, individuals with schizophrenia were almost three times as likely as those with drug or alcohol use disorders to be admitted to the hospital. Individuals with mood disorders were over four times as likely to be admitted. While substance use disorders accounted for over half of all behavioral health transports, a quarter of these transports resulted in hospital admission [10].

Despite increasing awareness of the problem, the empirical knowledge base regarding EMS transport and ED utilization among persons with behavioral health conditions remains underdeveloped and has numerous limitations, such as a lack of information on the individual’s previous psychiatric history, limited information on ED disposition, and a poor description of what is classified as a behavioral health call. Given this, Detroit Wayne Integrated Health Network (DWIHN) [11] and Detroit East Medical Control Authority (DEMCA) [12] collaborated to study the overlap between behavioral health history and EMS with goals of care coordination and improvement.

## Materials and methods

DWIHN’s Research Advisory Committee (RAC) reviewed and approved the study (approval number: 041122-1).

Data source

All EMS runs data comes from DEMCA which provides medical direction to 11 EMS agencies that have over 5,000 providers. As part of their services, they create operational protocols for the providers and provide education and training. Their geographic service areas include the city of Detroit, Highland Park, Harper Wood, Grosse Pointe Park, Grosse Pointe Farms, Grosse Pointe Woods, and the city of Grosse Pointe. In 2020, the public and private EMS providers responded to 142,040 calls, of which over 63,500 calls were for behavioral health reasons [11].

The source of DWIHN’s data is based on approximately 250,000 individuals’ demographic data in DWIHN’s Data Warehouse. This dataset includes members from 2010 to the present (July 2021) and does not exclude inactive members or those who registered but did not receive services.

DWIHN established a data-sharing agreement with DEMCA which also has data-sharing agreements with EMS partners to support continuity of care. DWIHN and EMS data were “fuzzy” matched, both exact word matches of names and dates of birth as well as semi-exact permutations of names. This technique allows for high-tolerance identification of the same person in both datasets. This match allowed insights into EMS use by the community mental health members of Wayne county. This helped us identify the overlapping population and the distribution of EMS runs in Detroit/Wayne County with more targeted information on these specific dual members. The data are confidential and only available to the staff involved in care. Deidentified data are shared during collaborative meetings.

## Results

**Figure 1:**
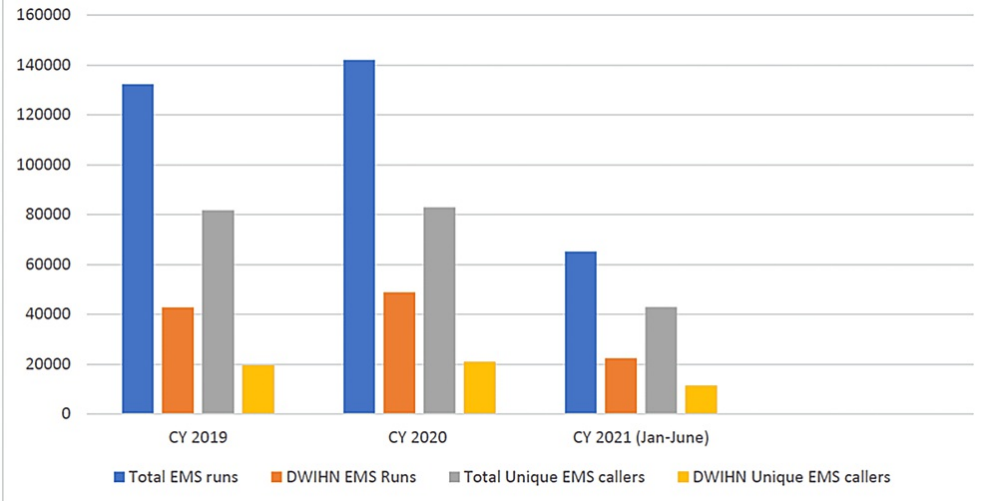
DWIHN-EMS data for 2019, 2020, and 2021 (January-June). CY: cumulative year DWIHN: Detroit Wayne Integrated Health Network; EMS: emergency medical service

During 2019, approximately one-third of EMS runs (32.4%) involved individuals served by DWIHN. This increased slightly to 34.4% in 2020 and has remained steady through June 2021. It was observed that some DWIHN members were requesting multiple EMS runs. For instance, in 2020, there were 20,934 DWIHN EMS users who requested 48,886 runs (Figure 1). People with repeated runs were recognized as “familiar faces” by DWIHN. DWIHN also identified common geographical locations where EMS was more frequently requested, as well as the identification of hospitals that received most of those runs. We were able to identify the common diagnosis that correlated with higher EMS runs and found the highest correlation with schizoaffective disorder, followed by schizophrenia and alcohol use disorder.

We reviewed more recent data from 2020 and January-June 2021 and noted that, of the total 71,283 DWIHN EMS runs during this time period, the most common first impressions of the calls were general malaise - unknown cause (n = 5,873), psychiatric/behavioral problem (n = 4,185), and no apparent illness or injury (n = 3,811).

## Discussion

With our data analysis, we found that a high number of EMS runs involved individuals with a behavioral health history, as indicated by their association with DWIHN. Of the 48,886 DWIHN EMS runs reported in 2020, nearly 43% (42.8%) involved repeat callers. In the first six months of 2021, that percentage rose to 50.8%. Consistent with previous findings [9,10], a diagnosis of schizophrenia spectrum disorders accounted for the highest number of EMS runs, followed by alcohol use disorder. Moreover, on pairing the datasets obtained from DWIHN and DEMCA, we identified the top DWIHN-associated repeat callers, providing a unique opportunity for current and future coordination of care.

Using the findings from the study, DWIHN developed the following two targeted interventions: (1) The zip codes of the EMS callers and hospitals are identified and used to organize mental health outreach and treatment events through mobile units. (2) DWIHN has developed a protocol for comprehensive case analyses of DWIHN-EMS familiar faces with an identified pathway for both medical and behavioral health callers. The repeated EMS callers are being referred to our complex case management for coordination of medical and psychiatric care. Further case discussions and clinical consultations are being coordinated with the member’s outpatient providers to discuss challenges with treatment recommendations, as well as discussions around consideration of more intensive behavioral health services.

Earlier studies have shown that EMS callers with behavioral health histories may be stigmatized and under-triaged [13]. As a result of this growing realization, EMS systems are beginning to institute policy and procedural changes to help EMS and first responders identify and respond appropriately to callers undergoing a behavioral health crisis [14]. Mental health clinicians are partnering with local police departments and EMS systems to design and implement crisis intervention training for police officers and first responders to help them determine if an incident involves behavioral health, apply appropriate de-escalation skills, and triage cases that require psychological intervention rather than making arrests and incarcerating the mentally ill [4,15].

In response to the rising need for integration with first responders, DWIHN has incorporated the following established, preventive strategies to reduce the number of recurrent behavioral health-related EMS calls, prevent the occurrence of such crises, and avoid unwarranted involvement with the justice system: (1) Expanded Crisis Intervention Team Training for first responders [16]: between August 2020 and September 2021, 198 individuals successfully completed training in the 40-hour CIT course for first responders. In the fiscal year 2021 (October 2020 to September 2021) alone, 161 individuals completed the course. (2) Collaborated with the Detroit Police Department to develop a three-prong pilot program pairing CIT-trained police officers or first responders with trained behavioral health clinicians [17]. The goals of this partnership are to provide: (A) support to CIT-trained officers responding to behavioral health calls, (B) referral to appropriate support services for callers experiencing a mental health crisis, (C) support to 911 call centers handling behavioral health crises, and (D) outreach to targeted “hot-spot” locations with the overarching goal of preventing justice system involvement among individuals experiencing behavioral health crises. The first approach pairs two CIT-trained officers with a behavioral health specialist to operate a specialized car that focuses on police responses to calls with a behavioral health nexus. Because trained clinicians are better able to identify and classify calls involving mental health crises, the second strategy includes an embedded 911 response with DWIHN clinicians at the communications call center who directly connect callers experiencing behavioral health emergencies to support services. The clinicians also assign calls for service to the CIT co-response units when appropriate. The embedded clinicians make follow-up calls to callers identified as high utilizers of 911 and connect them to mental health and other needed services. Finally, the teams of behavioral health specialists from DWIHN providers and CIT-trained officers patrol hot-spot locations, provide outreach services to those experiencing mental health and/or substance use problems, and help connect individuals to supportive services in addition to co-responding to police runs with a mental health nexus. The above programs are expected to provide support to 911 call centers that have shown to lack resources to handle behavioral health crises at this point [18].

Study limitations

The EMS data is for runs and not 911 calls and, therefore, indicate only a portion of the EMS contacts. Second, EMS data are for limited cities on the eastern side of Wayne County, and there is room for expanding it to capture the entire county that is served by DWIHN. Lastly, the EMS data capture the status of the member at the time of the EMS run, and the fuzzy match to DWIHN’s data is linked to the person, not to the claim. The DWIHN dataset dates back to 2010 and a person may have received behavioral health services at any point during that period. Therefore, time series comparisons may be non-parallel for some members who may not be active within the DWIHN network at the time of the EMS run or subsequently. Our study established a correlation between mental health history and psychiatric diagnoses with EMS use, but this needs to be further explored in randomized control studies. Our study was not able to look at the severity of psychiatric illness and EMS calls, and this can be investigated in future studies.

## Conclusions

With the implementation of multiple targeted and prevention interventions, we are hoping to see a reduction in the percentage of DWIHN-EMS callers and an improvement in the coordination of care. We plan to reanalyze EMS-DWIHN data in a year and readjust our interventions based on the findings. We suggest that other comparable organizations consider similar analysis and methodology, especially given the increasingly high EMS and ED use for behavioral health reasons.
